# Supplementary material for: Empirical evaluation of the association between daily living skills of adults with autism and parental caregiver burden
Source: PLoS One. 2021 Jan 5;16(1):e0244844. doi: 10.1371/journal.pone.0244844 (PMC7785247; doi:10.1371/journal.pone.0244844)
Supplement: S1 Fig — (DOCX) [file pone.0244844.s001.docx]

**Supplemental Figure 1. Correlations of caregiver burden with independent variables.**


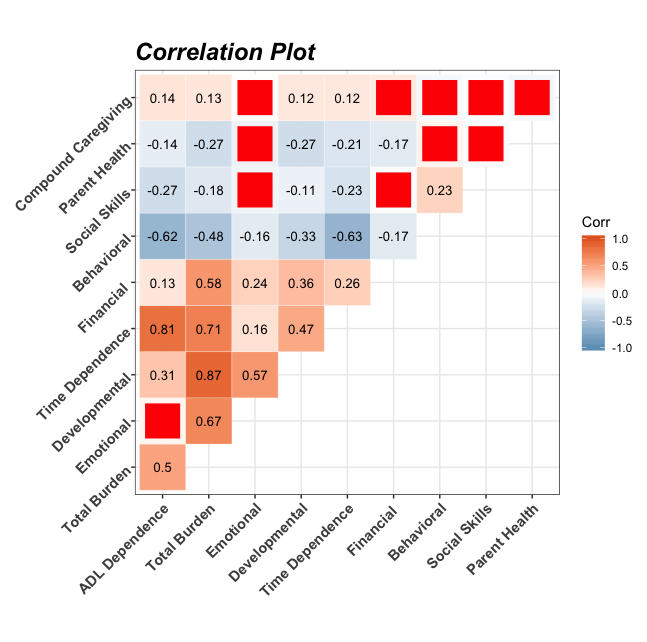


Note: Blue color indicates negative correlation. Orange color indicates positive correlation. Red solid squares mark non-statistically significant correlation between indicators. All other specified correlation in the matrix are significant at p<0.05.
