# Supplementary material for: Empirical evaluation of the association between daily living skills of adults with autism and parental caregiver burden
Source: PLoS One. 2021 Jan 5;16(1):e0244844. doi: 10.1371/journal.pone.0244844 (PMC7785247; doi:10.1371/journal.pone.0244844)
Supplement: S2 Fig — (DOCX) [file pone.0244844.s002.docx]

**Supplemental Figure 2: Linear, quadratic, and non-parametric (Lowess) representations of bivariate associations between ADL and caregiver burden.**


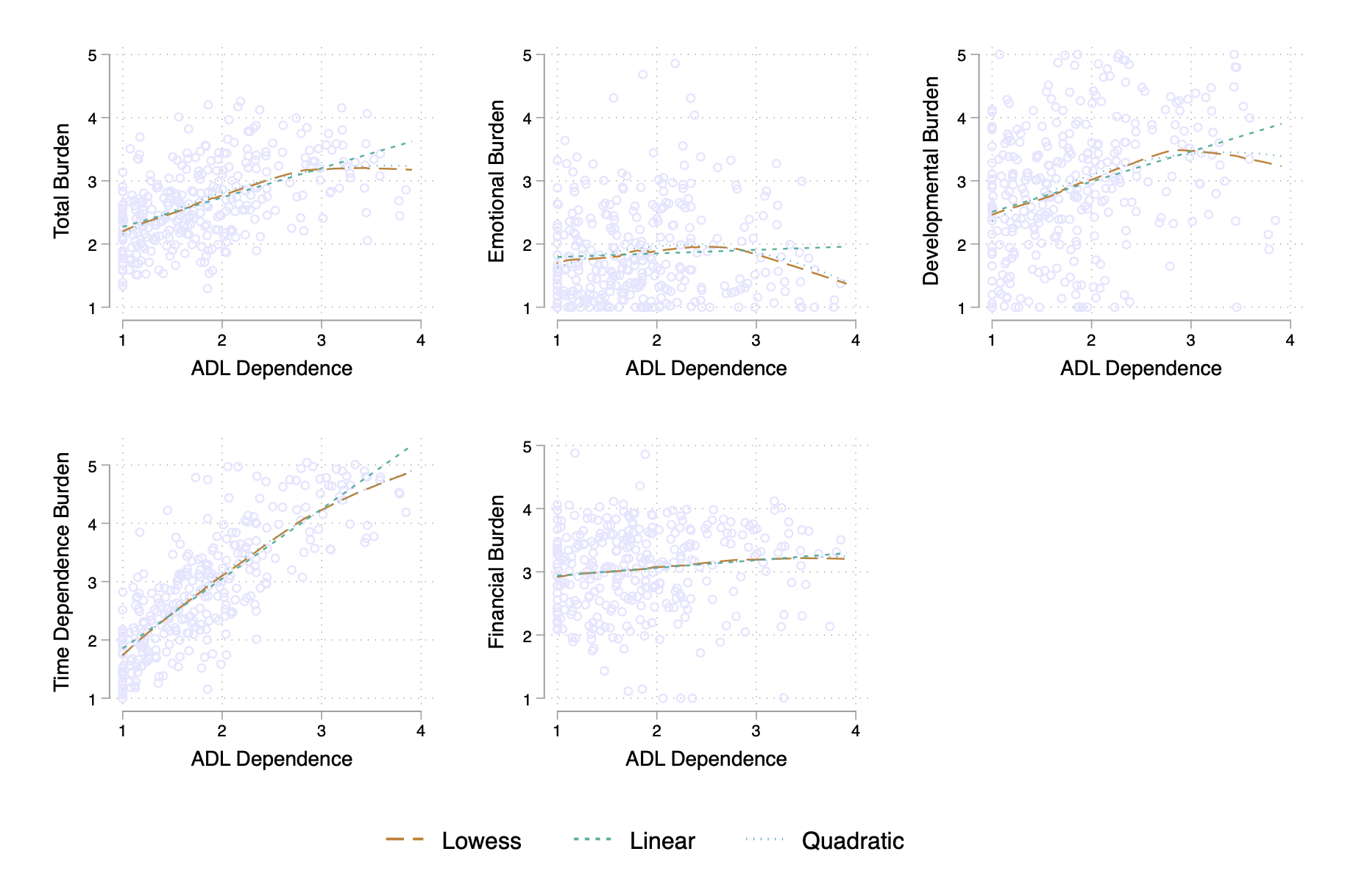


**Note:** scatter is displayed with a 4 points jitter.
