# Supplementary material for: Empirical evaluation of the association between daily living skills of adults with autism and parental caregiver burden
Source: PLoS One. 2021 Jan 5;16(1):e0244844. doi: 10.1371/journal.pone.0244844 (PMC7785247; doi:10.1371/journal.pone.0244844)
Supplement: S3 Fig — (DOCX) [file pone.0244844.s003.docx]

**Supplemental Figure 3. Linear associations between ADL and caregiver burden.**


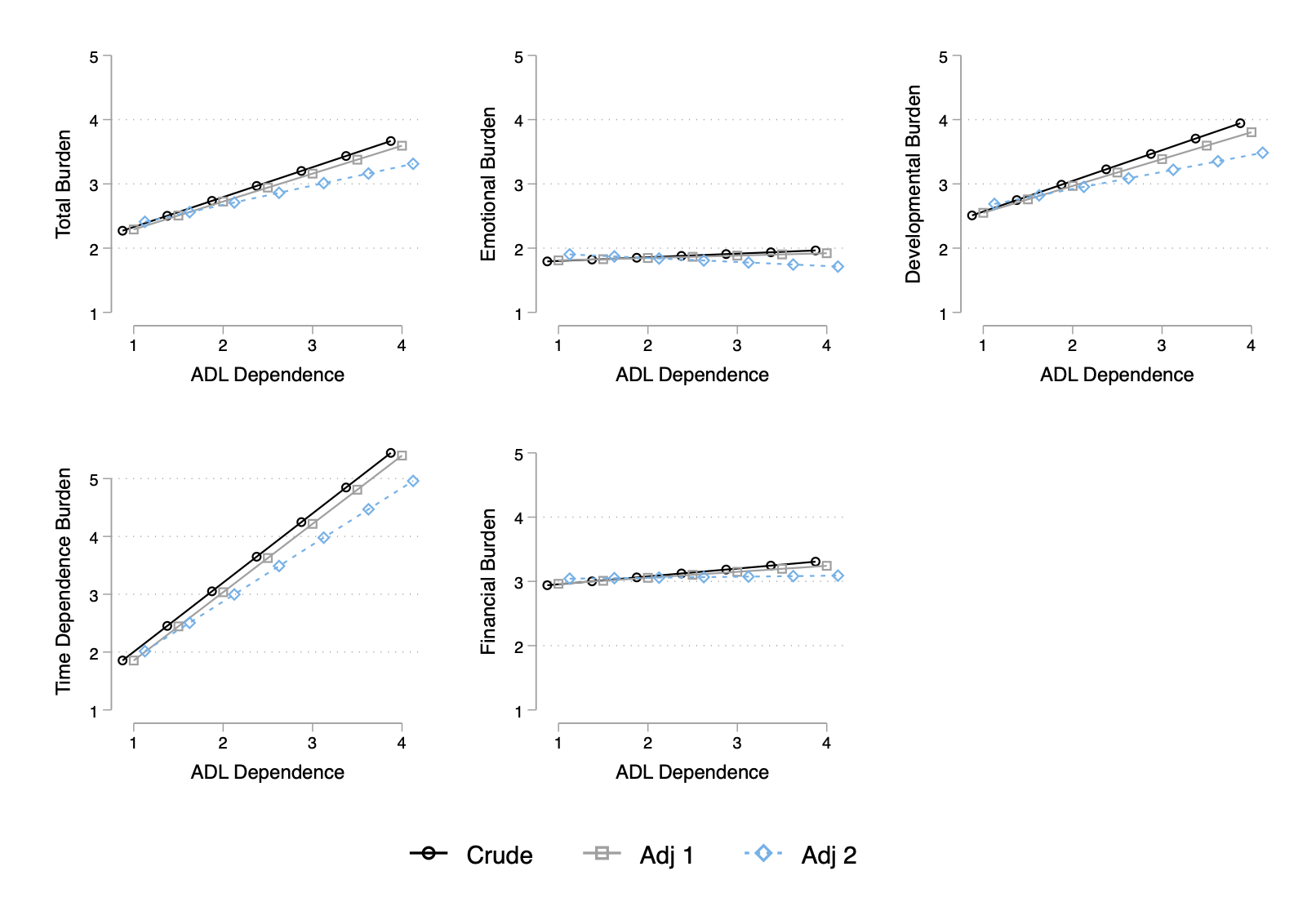


**Notes:**

1.M1: Crude; Adj1: M1 + caregiver characteristics; Adj2: Adj1 + child communication, behavioral and social characteristics.

2.Plots are based on marginal mean estimates derived from incrementally adjusted linear regression models.
