# Supplementary material for: Empirical evaluation of the association between daily living skills of adults with autism and parental caregiver burden
Source: PLoS One. 2021 Jan 5;16(1):e0244844. doi: 10.1371/journal.pone.0244844 (PMC7785247; doi:10.1371/journal.pone.0244844)
Supplement: S4 Fig — (DOCX) [file pone.0244844.s004.docx]

**Supplemental Figure 4: Linear, quadratic, and cubic associations between ADL and caregiver burden***.*


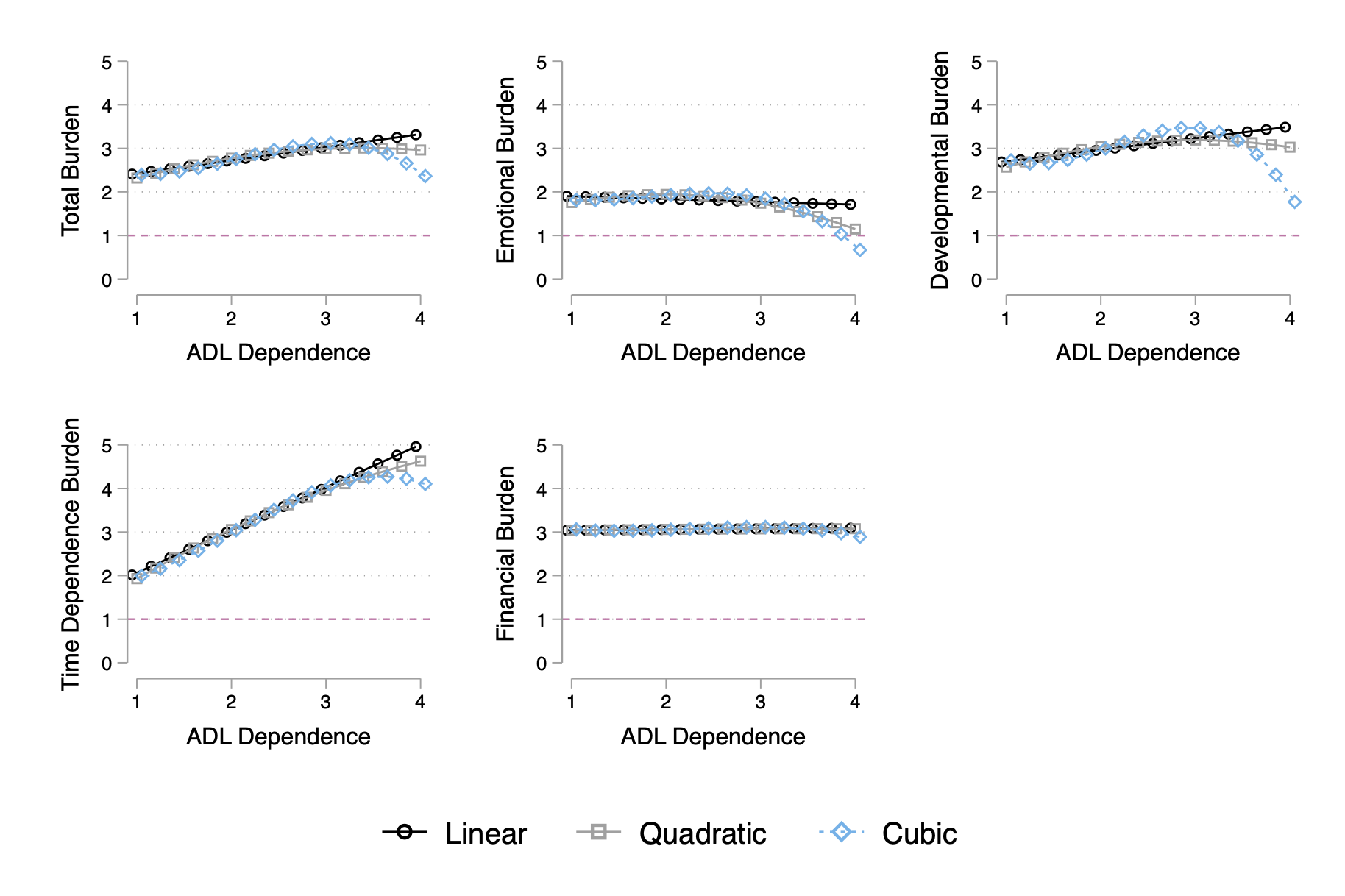


**Notes**:

1. Estimates derived from models adjusted for parent caregiver characteristics and adult child characteristics.

2. Plots are based on marginal mean estimates derived from fully adjusted linear regression models.
