# Supplementary material for: Empirical evaluation of the association between daily living skills of adults with autism and parental caregiver burden
Source: PLoS One. 2021 Jan 5;16(1):e0244844. doi: 10.1371/journal.pone.0244844 (PMC7785247; doi:10.1371/journal.pone.0244844)
Supplement: S5 Fig — (DOCX) [file pone.0244844.s005.docx]

**Supplemental Figure 5: Linear associations between ADL dependence and caregiver burden.**


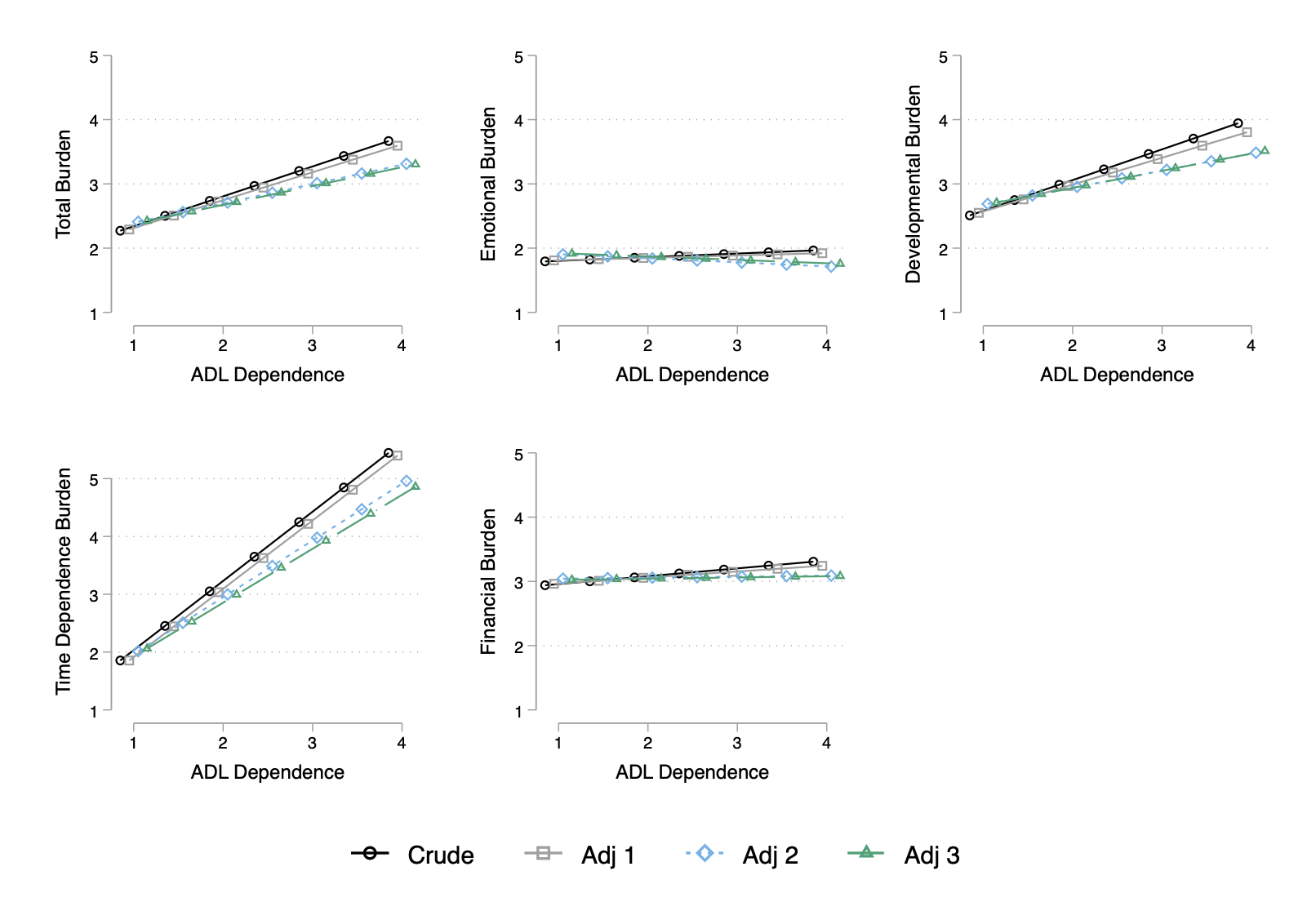


**Notes**:

1. M1: Crude; Adj1: M1 + parent caregiver characteristics; Adj2: Adj1 + adult child characteristics; Adj3: Adj2 + parent caregiver characteristics (age, gender, race, marital status, employment status, educational attainment, household income, caregiving responsibilities), adult child characteristics (gender, employment status, educational attainment, living arrangement), total number of children in the household, and total number of children with disabilities.
2. Plots are based on marginal mean estimates derived from incrementally adjusted linear regression models.
