# Supplementary material for: Empirical evaluation of the association between daily living skills of adults with autism and parental caregiver burden
Source: PLoS One. 2021 Jan 5;16(1):e0244844. doi: 10.1371/journal.pone.0244844 (PMC7785247; doi:10.1371/journal.pone.0244844)
Supplement: S1 Table — (DOCX) [file pone.0244844.s006.docx]

**Supplemental Table 1. Fit Statistics for alternative parametric fit of the association between ADL and caregiver burden outcomes.**

|  |  | **N** | **Null Log Likelihood** | **Alternative Log Likelihood** | **df** | **AIC** | **BIC** | **R^2^** |
| --- | --- | --- | --- | --- | --- | --- | --- | --- |
| **Total Burden** | |  |  |  |  |  |  |  |
|  | Linear | 303 | -293.7 | -221.2 | 11 | 464.5 | 505.3 | 0.38 |
|  | Quadratic | 303 | -293.7 | -218.8 | 12 | 461.6 | 506.2 | 0.39 |
|  | Cubic | 303 | -293.7 | -215.7 | 13 | 457.5 | 505.7 | 0.40 |
|  | Natural Log | 303 | -293.7 | -219.9 | 11 | 461.8 | 502.7 | 0.39 |
| **Emotional Burden** | |  |  |  |  |  |  |  |
|  | Linear | 303 | -350.6 | -334.2 | 11 | 690.4 | 731.3 | 0.10 |
|  | Quadratic | 303 | -350.6 | -331.2 | 12 | 686.4 | 731.0 | 0.12 |
|  | Cubic | 303 | -350.6 | -330.3 | 13 | 686.5 | 734.8 | 0.13 |
|  | Natural Log | 303 | -350.6 | -334.4 | 11 | 690.9 | 731.7 | 0.10 |
| **Developmental Burden** | | |  |  |  |  |  |  |
|  | Linear | 303 | -446.2 | -409.7 | 11 | 841.3 | 882.2 | 0.21 |
|  | Quadratic | 303 | -446.2 | -408.5 | 12 | 841.0 | 885.5 | 0.22 |
|  | Cubic | 303 | -446.2 | -404.6 | 13 | 835.2 | 883.5 | 0.24 |
|  | Natural Log | 303 | -446.2 | -409.3 | 11 | 840.5 | 881.4 | 0.22 |
| **Time Dependence Burden** | | |  |  |  |  |  |  |
|  | Linear | 303 | -432.2 | -235.3 | 11 | 492.5 | 533.4 | 0.73 |
|  | Quadratic | 303 | -432.2 | -233.2 | 12 | 490.5 | 535.1 | 0.73 |
|  | Cubic | 303 | -432.2 | -231.1 | 13 | 488.3 | 536.5 | 0.73 |
|  | Natural Log | 303 | -432.2 | -237.2 | 11 | 496.4 | 537.2 | 0.72 |
| **Financial Burden** | |  |  |  |  |  |  |  |
|  | Linear | 301 | -292.1 | -278.3 | 11 | 578.6 | 619.4 | 0.09 |
|  | Quadratic | 301 | -292.1 | -278.3 | 12 | 580.6 | 625.1 | 0.09 |
|  | Cubic | 301 | -292.1 | -278.1 | 13 | 582.2 | 630.4 | 0.09 |
|  | Natural Log | 301 | -292.1 | -278.3 | 11 | 578.6 | 619.4 | 0.09 |
