# Supplementary material for: Empirical evaluation of the association between daily living skills of adults with autism and parental caregiver burden
Source: PLoS One. 2021 Jan 5;16(1):e0244844. doi: 10.1371/journal.pone.0244844 (PMC7785247; doi:10.1371/journal.pone.0244844)
Supplement: S2 Table — (DOCX) [file pone.0244844.s007.docx]

**Supplemental Table 2. Alternative parametric associations, and fit statistics, between ADL and caregiver burden outcomes.**

|  | M1 | M2 | M3 | M4 |
| --- | --- | --- | --- | --- |
|  | b/se | b/se | b/se | b/se |
|  | **Total Burden** | | | |
| **Linear** | 0.301*** | 0.819** | -1.676 |  |
|  | 0.063 | 0.247 | 1.051 |  |
| **Quadratic** |  | -0.121* | 1.114* |  |
|  |  | 0.056 | 0.509 |  |
| **Cubic** |  |  | -0.186* |  |
|  |  |  | 0.076 |  |
| **Log** |  |  |  | 0.594*** |
|  |  |  |  | 0.117 |
| **R^2^** | 0.38 | 0.39 | 0.40 | 0.39 |
| **AIC** | 464.5 | 461.6 | 457.5 | 461.8 |
| **BIC** | 505.3 | 506.2 | 505.7 | 502.7 |
|  | **Emotional Burden** | | | |
| **Linear** | -0.063 | 0.775* | -1.227 |  |
|  | 0.091 | 0.358 | 1.533 |  |
| **Quadratic** |  | -0.195* | 0.795 |  |
|  |  | 0.081 | 0.742 |  |
| **Cubic** |  |  | -0.149 |  |
|  |  |  | 0.111 |  |
| **Log** |  |  |  | -0.023 |
|  |  |  |  | 0.170 |
| **R^2^** | 0.10 | 0.12 | 0.13 | 0.10 |
| **AIC** | 690.4 | 686.4 | 686.5 | 690.9 |
| **BIC** | 731.3 | 731.0 | 734.8 | 731.7 |
|  | **Developmental Burden** | | | |
| **Linear** | 0.266* | 0.945* | -4.290* |  |
|  | 0.117 | 0.463 | 1.960 |  |
| **Quadratic** |  | -0.158 | 2.432* |  |
|  |  | 0.104 | 0.949 |  |
| **Cubic** |  |  | -0.390** |  |
|  |  |  | 0.142 |  |
| **Log** |  |  |  | 0.534* |
|  |  |  |  | 0.218 |
| **R^2^** | 0.21 | 0.22 | 0.24 | 0.22 |
| **AIC** | 841.3 | 841.0 | 835.2 | 840.5 |
| **BIC** | 882.2 | 885.5 | 883.5 | 881.4 |
|  | **Time Dependence Burden** | | | |
| **Linear** | 0.981*** | 1.476*** | -0.698 |  |
|  | 0.066 | 0.259 | 1.105 |  |
| **Quadratic** |  | -0.115* | 0.960 |  |
|  |  | 0.059 | 0.535 |  |
| **Cubic** |  |  | -0.162* |  |
|  |  |  | 0.080 |  |
| **Log** |  |  |  | 1.824*** |
|  |  |  |  | 0.124 |
| **R^2^** | 0.73 | 0.73 | 0.73 | 0.72 |
| **AIC** | 492.5 | 490.5 | 488.3 | 496.4 |
| **BIC** | 533.4 | 535.1 | 536.5 | 537.2 |
|  | **Financial Burden** | | | |
| **Linear** | 0.016 | 0.034 | -0.756 |  |
|  | 0.076 | 0.305 | 1.310 |  |
| **Quadratic** |  | -0.004 | 0.386 |  |
|  |  | 0.069 | 0.633 |  |
| **Cubic** |  |  | -0.059 |  |
|  |  |  | 0.095 |  |
| **Log** |  |  |  | 0.027 |
|  |  |  |  | 0.143 |
| **R^2^** | 0.09 | 0.09 | 0.09 | 0.09 |
| **AIC** | 578.6 | 580.6 | 582.2 | 578.6 |
| **BIC** | 619.4 | 625.1 | 630.4 | 619.4 |

Notes: Results are based on linear regression models with full adjustments for covariates. M1: Linear; M2: Quadratic; M3: Cubic, M4: Natural log transformation.

*p<0.05; **p<0.01; ***p<0.001
