# Supplementary material for: Empirical evaluation of the association between daily living skills of adults with autism and parental caregiver burden
Source: PLoS One. 2021 Jan 5;16(1):e0244844. doi: 10.1371/journal.pone.0244844 (PMC7785247; doi:10.1371/journal.pone.0244844)
Supplement: Attachment — Submitted filename: Response to reviewers PlosOne July 16 2020.docx [file pone.0244844.s010.docx]

**Response to reviewers: PLOS one**

|  | **Reviewer/ Editor suggestion or critique** | **Authors’ response** |
| --- | --- | --- |
| **R1.1** | One limitation of the study is the extremely **modest contribution to the extant literature**. The primary finding is that lower adaptive functioning in daily living of individuals with autism was associated with greater perceived caregiver burden. This is finding is well-established across the life course in this population specifically and more broadly with parental caregivers of adults with disabilities. There is a well-established research literature spanning 40 years on Community Living Opportunities for persons with disabilities. It would be helpful to incorporate at least a brief review of this literature into the introduction. After incorporating the above foundation, it would be helpful to consider and state the relevance of the findings relative to the literature above. | Thank you for raising this concern. Our systematic search for literature that establishes the association of caregiver burden with ADL status of adults with ASD was limited. We added references from other disabilities and stated on pages 3-4:  Much of the evidence establishing the association of caregiving burden with functional independence in caregiving literature is based on research conducted among adult-onset conditions such as stroke and dementia(15,16) and unspecified intellectual/ developmental disabilities(17,18). In comparison, there is sparse research on the dynamic association between caregiving burden and functional independence of children with ASD, particularly among adolescents and adults (19–21).  Then again on page 6 we also stated: “Although it is well-established that children with ASD benefit from interventions targeted to improve their daily living skills(45–48), there is a paucity of interventions focused on individuals with ASD across the lifespan and their family members (49,50).  So, although we agree with Reviewer 1 that there is a body of literature focused on the topics on adaptive skills of poor parental well-being, we find that there is a dearth of such literature among adults with ASD and their aging parents. Therefore, we have rewritten sections of the introduction after reviewing the literature We now hope that the contribution of this paper to disability literature by linking ADL status of adults with ASD with caregiver burden is now clearer. |
| **R1.2** | One limitation of the literature review is its **conceptualization of ADL** dependence as primarily an intraindividual phenomenon without attention to or coverage of the **well-established literature on the crucial role of intervention and supports preceding, during, and following the transition to adulthood for persons with disabilities** and specifically with autism. In fact, the lack of such supports is a major contributing factor to the lack of development of independent ADL, which preclude opportunities for independent living. | We have rewritten the sections pertaining to conceptualization of ADL. Thank you for this suggestion |
| **R1.3** | Measures and analytical approach are appropriate to address the research questions. A limitation of the sampling is that it **overrepresents white, higher income** families and yet we know that the systemic inequities place individuals who are of non-dominant race and/or who are socioeconomically disadvantaged at higher risk for developmental disabilities.   a. The sampling limitations noted above should be noted as limitations in the Discussion section. | We agree this is now emphasized in the limitations section.  “The overrepresentation of non-Latino white families from a higher socioeconomic status limits the generalizability of our findings. Future research should include a broader array of aging parents of adults with ASD, particularly by targeting the recruitment of racial/ ethnic minorities and those living in low resource disadvantaged households and communities.” |
| **R2.1** | Weak links in the justification for the study. This was notable in the Abstract and also in the beginning of the introduction. By the end of the introduction, these links were made, but I believe edits to the abstract and beginning of the introduction could greatly strengthen the manuscript. I've further described these in two bullets below.   a. In the **abstract**, the second sentence did not adequately link from the first sentence. It does not read as a clear "Therefore" between these two ideas. It felt like a leap when reading it on its own. b. In the **introductory** **section**, I found similar issues with leaps in reasoning that don't quite link. The second paragraph on page 3 starts with a sentence that begins stating that there is a benefit, but then finishes stating that there are detrimental impacts on health. Those detrimental impacts are not described, and instead the next sentence begins to talk about more benefits. The reasoning here was hard to follow. Next, there is a sentence that again makes a big leap "parenting can be bittersweet" and then specifically states the study purpose. I believe there is a need for more clearer and stronger writing in this paragraph to more clearly draw these connections and build the rationale for this project. Of course you provide more information alter in the introduction (which was done effectively), but this paragraph as it is currently written does not work. | Thank you for pointing out this lack of flow. We have rewritten these sections carefully again. |
| **R2.2** | Second, I suggest that the authors use the acronym "ADLs" rather than "ADL" for activities of daily living. This will make its representation of a plural much more clear, and is consistent with how AOTA uses this term (see the OTPF). When making this change, the authors should review every sentence in which it is used to make sure there is subject-verb agreement, as this was somewhat inconsistent throughout the paper (currently ADL is used as both a singular and plural noun at different points). For example, the phrase "ADL status" would sound strange as "ADLs status" so those instances should be edited throughout ("independence in ADLs" may be a reasonable edit). | Thank you for pointing this out. We have checked and edited this word throughout the paper as dependence / independence in ADLs |
| **R2.3** | A**nalyses/results**, there is some lack of clarity. How were the correlations run (Pearson? Spearman? some variables were linear and others were considered nominal?)? More importantly, I don't actually view the correlations as useful to include. Many of the magnitudes are incredibly small (many 0.1 and 0.2), yet interpreted as "significant". (Magnitudes are stated to be "small" for emotional and financial burden, but there are many others, for example all were small for compound caregiving). They are also not considered further at all in the paper. I believe it would be sufficient to just state that you examined correlations prior to running the regression models. I didn't find that they added anything to the paper. The figure for them was also a bit confusing with the use of the red square for non-significant correlations (especially when alongside dark orange representing strong positive correlations) and that the x and y axes were not in a consistent order. For the regression results, "beta" is reported but it is not clear whether they are standardized or unstandardized coefficients. I also did not find the regression figures to be critical for the paper. Currently, they seem to be duplicated in the supplemental files (in main paper and supplemental). I feel they could work fine as just supplemental and not in the main paper. | (1) We placed the correlation plot as a Supplemental Figure 1. (2) The unstandardized beta coefficients estimated through the regression models are now clearly stated in text. (3) Regression figures are now included as supplements.   References to all figures have been updated in text. |
| **R2.4** | **Discussion section**, there are some points that could be made more effectively. Currently, there is an implication that interventions should be provided to adolescents and adults on the spectrum ONLY for the purpose of reducing caregiver burden. I recognize that this ties best with your results, but it comes across strangely and I believe it would be much more appropriate to also discuss the potential benefits of this for the individuals themselves (there is available literature on this). | We have rewritten and included references on the individual-level benefits of ASD though it was not the primary focus on this paper. |
| **R2.5** | In the **limitations section**, the authors state that their lack of diversity is acceptable because they were able to control for participant characteristics. However, this is misleading, since one cannot truly control for things when there is high heterogeneity in the sample. This should be re-stated. | We agree, we took this out stated this as a study limitation per the reviewer’s recommendation. The limitations section was adjusted accordingly.  ~~Despite the lack of participant diversity, the large sample size enabled us to control for the caregiving related characteristics of the parent and adult child.~~  “The overrepresentation of non-Latino white families from a higher socioeconomic status limits the generalizability of our findings. Future research should include a broader array of aging parents of adults with ASD, particularly by targeting the recruitment of racial/ ethnic minorities and those living in low resource disadvantaged households and communities.”. |
| **R2.6** | On page 5, the term "independent living" is used, where I believe what is meant is "independence in ADLs" (these are not interchangeable terms): | Thank you for pointing this out. We have rewritten the sentence.  “Importantly, adults with ASD were unable to complete more than 30% of their ADLs independently. These findings highlight the critical need for ongoing support in adulthood to maintain independence in BADLs and gain independence in IADLs”. |
| **R2.7** | On page 7, when describing participant ages, 60 is included in both categories. This should be edited to indicate either 50-59 or 61+, depending on which category 60 year olds were considered in. The majority of the participants (*n* = 212, 66.3%) were aged 50-60 years, while the remaining n =108 (33.7%) were aged 60-years or older. | This was corrected in the text and the Table. The categories are 50-59 and 60+ years. |
| **R2.8** | Page 7, typo: "bring" should be "being" | Yes, this was corrected. Thank you. |
| **R2.9** | Page 12, where it says "group (3)" I believe it would be more clear to say "a score of 3" | We have revised the text to specify the exact group that was used as the referent.  ~~This indicator was included as a nominal variable in the model using group (3) as the reference category.~~  The revised sentence reads as follows:  “This indicator was included as a nominal variable in the model using “Communicate needs and wants in a meaningful way” as the reference category”. |
| **R2.10** | Sentence spanning pages 13-14 was long and hard to read-suggest editing: | We agree. The sentence was revised for better style and more clarity.  *Plots representing the crude, and incrementally adjusted estimates of marginal means of each of the considered burden outcomes over the ADL continuum, and their 95% confidence intervals, are presented in Figure 2 to facilitate the visualization of the reported associations and reductions in magnitudes resulting from covariates adjustments (39):*  “The marginal means plots for each of the burden outcomes over the ADL dependence continuum, and their 95% confidence intervals, are presented in Supplemental Figure 3. These plots help visualize the reported associations and the reductions in magnitudes resulting from covariates adjustments”(50). |
| **R2.11** | In table 2, I believe you should remove the decimal points in the row reporting the sample size: | Yes, this was corrected. Thank you. |
| **R2.12** | page 21, "emerging growing" --only one of these words is needed | Yes, this was corrected. Thank you. |
| **R2.13** | page 24, "minorities" may be better stated as "racial/ethnic minorities" | Yes, this was corrected. Thank you. |
| **E1** | Please ensure that your manuscript meets PLOS ONE's **style requirements**, including those for file naming using the The PLOS ONE style templates | Yes, they have been checked. |
| **E2** | Please include additional information regarding the survey or questionnaire used in the study and ensure that you have provided sufficient details that others could replicate the analyses. For instance, if you developed a **questionnaire** as part of this study and it is not under a copyright more restrictive than CC-BY, please include a copy, in both the original language and English, as Supporting Information. | We have now cited the dissertation report which included the survey that is published and available online in digital commons (accessible through a library system). We can also upload a copy of the survey if this citation is not sufficient for the reader. |
| **E3** | Please refer to any **sample size calculations** performed prior to participant recruitment. If these were not performed please justify the reasons. Please refer to our statistical reporting guidelines for assistance (https://journals.plos.org/plosone/s/submission-guidelines.#loc-statistical-reporting). | We added the following sentences to the research design section in Methods  “Power analysis using G*Power 3.1(57) was undertaken before starting data collection to determine the appropriate sample size. The results indicated that we would need a sample of 242 participants to conduct multiple regression analyses with six independent variables, using blocks, at an alpha level of 0.05, and a power of 0.80”. |
| **E4** | Thank you for stating the following in the Acknowledgments Section of your manuscript: "This research was funded in part by dissertation research funding from Wayne State University’s Graduate School and the School of Social Work that enabled the data collection." We note that you have provided funding information that is not currently declared in your Funding Statement. However, funding information should not appear in the Acknowledgments section or other areas of your manuscript. We will only publish funding information present in the **Funding Statement section of the online submission form**. Please remove any funding-related text from the manuscript and let us know how you would like to update your Funding Statement. Currently, your Funding Statement reads as follows: "The funders had no role in study design, data collection and analysis, decision to publish, or preparation of the manuscript." | We have now fixed this. |
| **E5** | Thank you for stating the following in your Competing Interests section: "No." Please complete your **Competing Interests on the online submission form** to state any Competing Interests. If you have no competing interests, please state "The authors have declared that no competing interests exist.", as detailed online in our guide for authors at [http://journals.plos.org/plosone/s/submit-now](about:blank) This information should be included in your cover letter; we will change the online submission form on your behalf. | We have now filled out this form. |
| **E6** | In your Data Availability statement, you have not specified where **the minimal data set** underlying the results described in your manuscript can be found. PLOS defines a study's minimal data set as the underlying data used to reach the conclusions drawn in the manuscript and any additional data required to replicate the reported study findings in their entirety. All PLOS journals require that the minimal data set be made fully available. For more information about our data policy, please see <http://journals.plos.org/plosone/s/data-availability>.  Important: If there are ethical or legal restrictions to sharing your data publicly, please explain these restrictions in detail. Please see our guidelines for more information on what we consider unacceptable restrictions to publicly sharing data: http://journals.plos.org/plosone/s/data-availability#loc-unacceptable-data-access-restrictions. Note that it is not acceptable for the authors to be the sole named individuals responsible for ensuring data access.  We will update your Data Availability statement to reflect the information you provide in your cover letter. | Data set is now uploaded as supplemental information |
| **E7** | Please include **captions for your Supporting Information files** at the end of your manuscript, and update any in-text citations to match accordingly. Please see our Supporting Information guidelines for more information: [http://journals.plos.org/plosone/s/supporting-information](about:blank). | We listed the files to match in-text citations. |
