# Supplementary material for: Empirical evaluation of the association between daily living skills of adults with autism and parental caregiver burden
Source: PLoS One. 2021 Jan 5;16(1):e0244844. doi: 10.1371/journal.pone.0244844 (PMC7785247; doi:10.1371/journal.pone.0244844)
Supplement: Attachment — Submitted filename: PLosOne response to reviewers 3 Nov 12_2020.docx [file pone.0244844.s011.docx]

**Response to PlosOne reviewers at round 3 of reviews**

|  | **Reviewer comments** | **Author response** |
| --- | --- | --- |
| R2+ | I found the updated version of this manuscript much improved. The writing was very clear and flowed well. The updates throughout made sense and helped increase clarity. | Thank you |
| R3+ | I appreciate that the authors commented on positives as well as negatives of caring for children on the autism spectrum. I am also pleased to see the recognition of ongoing parental support because this is often neglected. Conclusion: this is nice and clear | Thank you |
| R2.1 | Please clarify why 50 years of age was selected as the minimum age for participants. | Under the header “**Participants**,” clarification regarding the age of eligibility for this study has been added. Specifically, the following sentence was added:  “he age criterion of at least 50 years was selected to obtain information from an understudied population, aging parental caregivers of adult children with ASD. The specific age of 50 years was used as this age coincides with the age of enrollment for the American Association of Retired Persons. |
| R2.2 | There are still some issues with demographic response categories. See Table 1. ">60" years should be replaced with the 'greater than or equal to' sign. Similarly, Income levels I believe should be 'less than or equal to $40,000" (or use the symbol) since the next category starts at $40,001. | Thank you for pointing that mistake out. We fixed them as follows:   1. Changed to 60 years or above 2. Changed “Less than $40,000” to “$40,000 or below” |
| R2.3 | I find issues with the questions/wording used for the ASD characteristics. Most notably, the response items are not necessarily mutually exclusive. I understand that this was the tool used in the study, so it cannot be changed at this point, but problems with that measurement should be included as a limitation. It should also be noted in the measurement section that these items were developed by your research team (I assume?). | Yes, we have made the following additions to the manuscript to address this concern.  First, in the **Methods** section when describing the scales, we added the sentence: Each of these scales were developed for this study based on literature review.  Next, in the **limitations** section we added: The response categories used for determining the core characteristics of ASD were based on salient criteria available in the literature. The survey design, allowed respondents to choose only one criterion. However, it is possible for individuals to have a range of traits that overlap several responses.  And finally, in the **future directions** section, we added: Second, researcher-developed survey items included in this study could be refined by asking the participants to rank (on a scale of 0-100) the challenges of their adult child with ASD on each of the response categories within each of the domains (communication, social skills, and behavioral challenges). Doing so, would allow for a more precise characterization and adjustment to these challenges in statistical models. |
| R2.4 | I appreciate the clarity on the use of the communication scale as nominal and the use of "communicates needs and wants in a meaningful way" as the reference category. However, please edit so that both mentions of that response option are accurate. In the sentence describing the scale, the word "some" is included and then it is not included when it is indicated as the reference category. Use the exact language that was presented to participants. | We have now fixed the category labels in both tables. |
| R2.5 | The correlational analyses are not described in the data analysis section (but should be if they are included in the results section) | We think the reviewer missed reading our description of the correlational analyses. In the current text we state (in the 2^nd^ and 3^rd^ sentences of the data analysis section), that is pasted below:  “Second, prior to running regression models, pairwise correlation analyses were used to examine the bivariate relationships between the independent and dependent variables. Correlation plots were generated (Supplemental Figure 1) using the R software package(60,61).” |
| R2.6 | on page 21, the word "of" seemed missing in the phrase "availability medical insurance" | Thank you for pointing out this oversight. The typo is now fixed. |
| R3.1 | The adults that I work with prefer to use identity-first language rather than person-first. I therefore refer to my participants as autistic adults, please review the article by Lorcan Kenny (https://doi.org/10.1177/1362361315588200). | Yes, we recognize and are aware in practice and policy research that self-advocates prefer the use of the term “autistic”. However, as rightly pointed out in the article by Kenny et al (2015) it is still a grey area with deeply entrenched arguments.  Given that this paper was a structured interview that used person-first language in all data gathering tools to gather data from aging family caregivers, we prefer to use of person-first language in reporting our findings in this paper. |
| R3.2 | When writing I prefer not to start sentences with acronyms, on page 5 the authors do this and I would encourage them to reconsider this. I also prefer scientific writing to use the third-person point of view. However, these are stylistic comments and would not preclude publication. | We have removed acronyms from start of any sentence and revised the paper to remove all mention of first person and converted it all to the third person. |
| R3.3 | Introduction: The sentence "As individuals with ASD reach adulthood, many continue to require some form of reliance on their parents" needs to reviewed. It does not make sense to "require" "reliance" of someone. | The sentences were revised as follows:  “As individuals with ASD reach adulthood, many continue to need some form of assistance from their parents" |
| R3.4 | In the last sentence on page 4 the authors start a sentence with "because" and this is not an appropriate fashion to start sentences in academic writing - please review. | Parents often have to reduce working hours, forgo promotions, and higher educational pursuits, because of caregiving demands that can affect family income and retirement benefits (30,31). |
| R3.5 | On page 5, the sentence "Aging parents, who are themselves experiencing age-related health declines while supporting their adult children ..." needs reviewing. Please consider a different phrasing for "health declines". | Decline was replaced with the word challenges: "Aging parents, who are themselves experiencing age-related health challenges while supporting their adult children ..." |
| R3.6 | The authors state the purpose of the study twice. Is this necessary? | We have revised and deleted the first mention of the study purpose earlier in the literature review. |
| R3.7 | **Research design** - have the authors been provided with an ethics approval code? If so please include this code. | We have revised the text to include it in the manuscript: Approval was obtained from Wayne State University’s Institutional Review Board (IRB# 012615B3X) prior to beginning data collection. |
| R3.8 | The authors indicated that 353 surveys were received, but how many potential participants were provided with information regarding the survey. This would inform the reader of your response rate. | We could not determine the response rate, as we have no way of determining how many potential participants heard about the survey study. The survey link was sent out nationwide via newsletter, email, paper flyer, word of mouth, social media, etc. We present the details of how many attempted to complete the online survey and how some responses were excluded based on inclusion criteria. We have now included the following sentence in the limitations section: The survey response rate could not be determined, as there was no way of determining how many potential participants heard about the opportunity to participate given the wide spread methods of disseminating the survey link |
| R3.9 | Materials used - are the CBI and the CRA valid and reliable to use in this population. | No, there are no studies to date that have established the reliability/ validity of these tools in this population, but included in the manuscript were details about the validity of the tools for caregivers of other aging conditions such as dementia and how we adapted the tool for this population.  There is clearly a need to validate these tools for our target population and therefore we added the following sentence along with **future directions** of research: Finally, the next steps should include validation of the CBI and the CRA for aging caregivers of adults with ASD. |
| R3.10 | The authors have not included a "procedure" section. This information is included in other aspects of the manuscript, but it would be clearer if there was a stand-alone procedure section. | Thank you. This section is now created in the revised manuscript. |
| R3.11 | On page 21 on line 7, the authors switch to present tense when describing their results. Please change this to past tense. | We changed the sentence that read as “*Third*, our findings indicate that…”  To: *Third*, the findings indicated that… |
| R3.12 | **Discussion**: In the first paragraph of the discussion the authors indicated that future qualitative research will be completed. I feel this would be more appropriate to include in the 'future research' section of the discussion (just before the conclusion). | Thank you for bringing this to our attention. The qualitative research findings that were discussed were not based on “future qualitative research”, rather illustrates published findings based on a subset of the study sample. In the previous review, a reviewer indicated the need to include positive/ rewarding findings. As such, these details have been included here. Also, we moved this information towards the end of the discussion section (just before practice implications), to improve the flow of writing. |
| R3:13 | The first section of the discussion (before practice implications) initially describes the results and then link this to other results. I feel that this could be approached from a more integrated fashion, but I feel this is stylistic. | Thank you for the suggestion. We did reread and made a few adjustments to make the flow better. We believe the paper reads better now. |
